# Low level of Fibrillarin, a ribosome biogenesis factor, is a new independent marker of poor outcome in breast cancer

**DOI:** 10.1186/s12885-022-09552-x

**Published:** 2022-05-11

**Authors:** Flora Nguyen Van Long, Audrey Lardy-Cleaud, Dimitri Carène, Caroline Rossoni, Frédéric Catez, Paul Rollet, Nathalie Pion, Déborah Monchiet, Agathe Dolbeau, Marjorie Martin, Valentin Simioni, Susan Bray, Doris Le Beherec, Fernanda Mosele, Ibrahim Bouakka, Amélie Colombe-Vermorel, Laetitia Odeyer, Alexandra Diot, Lee B. Jordan, Alastair M. Thompson, Françoise Jamen, Thierry Dubois, Sylvie Chabaud, Stefan Michiels, Isabelle Treilleux, Jean-Christophe Bourdon, David Pérol, Alain Puisieux, Fabrice André, Jean-Jacques Diaz, Virginie Marcel

**Affiliations:** 1 Cancer Research Center of Lyon, Université Claude Bernard Lyon 1, INSERM 1052, CNRS 5286, Léon Bérard Cancer Centre, Cheney A, 28 rue Laennec, 69373 cedex 08 Lyon, France; 2 Institut Convergence PLAsCAN, 69373 cedex 08 Lyon, France; 3 DevWeCan Labex Laboratory, 69373 cedex 08 Lyon, France; 4 Biostatistics Unit, Department of Clinical Research, Léon Bérard Cancer Centre, 28 rue Laennec, 69008 Lyon, France; 5 Predictive Biomarkers and Novel Therapeutic Strategies Group, Institut Gustave Roussy, University of Paris Sud, INSERM 981, Université Paris Saclay, 114 rue Edouard Vaillant, 94800 Villejuif, France; 6 Department of Biostatistics and Epidemiology, Institut Gustave Roussy, 94800 Villejuif, France; 7 Tayside Tissue Bank, Ninewells Hospital and Medical School, NHS Tayside, Dundee, DD1 9SY Scotland UK; 8 Department Translational Research, Institut Gustave Roussy, 94800 Villejuif, France; 9 Department of Translational Research and Innovation, Léon Bérard Cancer Centre, 28 rue Laennec, 69008 Lyon, France; 10 Division of Cancer Research, University of Dundee, Ninewells Hospital and Medical School, Dundee, DD1 9SY Scotland UK; 11 Department of Pathology, University of Dundee, Ninewells Hospital and Medical School, Dundee, DD1 9SY Scotland UK; 12 Olga Keith Wiess Chair of Surgery, Dan L. Duncan Breast Center, Division of Surgical Oncology, Baylor College of Medicine, Houston, TX 77030 USA; 13 Université Paris-Saclay Institute of Neuroscience, CNRS UMR9197, Gif-sur-Yvette, France; 14 Université Paris-Saclay, CIAMS, 91405 Orsay, Cedex France; 15 Breast Cancer Biology Group, Translational Research Department, Institut Curie-PSL Research University, 26 rue d’Ulm, 75005 Paris, France

**Keywords:** Fibrillarin, Ribosome biogenesis, rRNA 2’O-ribose methylation complex, AgNOR, Breast cancer

## Abstract

**Background:**

A current critical need remains in the identification of prognostic and predictive markers in early breast cancer. It appears that a distinctive trait of cancer cells is their addiction to hyperactivation of ribosome biogenesis. Thus, ribosome biogenesis might be an innovative source of biomarkers that remains to be evaluated.

**Methods:**

Here, *fibrillarin* (*FBL*) was used as a surrogate marker of ribosome biogenesis due to its essential role in the early steps of ribosome biogenesis and its association with poor prognosis in breast cancer when overexpressed. Using 3,275 non-metastatic primary breast tumors, we analysed *FBL* mRNA expression levels and protein nucleolar organisation. Usage of TCGA dataset allowed transcriptomic comparison between the different *FBL* expression levels-related breast tumours.

**Results:**

We unexpectedly discovered that in addition to breast tumours expressing high level of *FBL*, about 10% of the breast tumors express low level of *FBL*. A correlation between low *FBL* mRNA level and lack of FBL detection at protein level using immunohistochemistry was observed. Interestingly, multivariate analyses revealed that these low *FBL* tumors displayed poor outcome compared to current clinical gold standards. Transcriptomic data revealed that *FBL* expression is proportionally associated with distinct amount of ribosomes, low *FBL* level being associated with low amount of ribosomes. Moreover, the molecular programs supported by low and high *FBL* expressing tumors were distinct.

**Conclusion:**

Altogether, we identified *FBL* as a powerful ribosome biogenesis-related independent marker of breast cancer outcome. Surprisingly we unveil a dual association of the ribosome biogenesis *FBL* factor with prognosis. These data suggest that hyper- but also hypo-activation of ribosome biogenesis are molecular traits of distinct tumors.

**Supplementary Information:**

The online version contains supplementary material available at 10.1186/s12885-022-09552-x.

## Background

Several studies have reported that increased protein synthesis induced by hyperactivation of ribosome biogenesis which occurs mainly within nucleoli, contributes to tumorigenesis by sustaining the hyperproliferative rate of cancer cells [1, 2]. The addiction of cancer cells to ribosome biogenesis hyperactivation is clearly illustrated by the numerous molecules developed in the last few years as cancer treatments that impair ribosome production either directly or indirectly [2–5]. Indeed, it has recently been shown that targeting ribosome biogenesis specifically kills cancer cells without affecting healthy ones [6, 7]. Moreover, a recent study revealed that oxaliplatin, conversely to other platinum-derived compounds, displays an anti-cancer activity through a ribosome biogenesis-dependent mechanism rather than through a DNA-damage response mechanism [8]. As a consequence, sensitivity of cancer cells to oxaliplatin is strongly correlated with expression levels of the different components making up the translation machinery [8]. Hyperactivation of ribosome biogenesis is a well-known marker of cancer cells [9]. Indeed, AgNOR staining (Argyrophillic Nucleolar Organiser region), which corresponds to silver staining of nucleolar regions where ribosome biogenesis takes place, is correlated with neoplastic transformation and cancer aggressiveness [9]. However, since such a correlation is not systematic, in particular in melanoma or mesothelioma [9], and automatic AgNOR staining and reading is still difficult to perform [10], this marker has never been approved for clinical purposes.

The ribosomal RNA (rRNA) methyltransferase fibrillarin (FBL) is one of the most abundant proteins present in nucleoli. This protein works as complex in concert with three proteins (NOP56, NOP58, NHP2L1) and exhibits several functional features, which are instrumental for ribosome biogenesis. On the one hand, FBL is one of the main regulators of several early steps of ribosome biogenesis, including ribosomal DNA (rDNA) synthesis and pre-rRNA cleavages [11–14]. On the other hand, FBL catalyzes the rRNA 2’-O-ribose methylation (2’-O-Me). It has also been reported that *FBL* expression is enhanced in prostatic neoplasia, in hepatocellular carcinoma and during mammary tumorigenesis [15–18]. In particular, tumors expressing high *FBL* levels are associated with poor outcome in breast cancer [15]. Moreover, we showed that *FBL* overexpression in MCF7 breast cancer cell lines promotes cell proliferation, colony formation and resistance to doxorubicin [15]. Indeed, we recently reported that alteration of *FBL* expression induces modulation of rRNA 2’-O-Me and directly affects translational activities of ribosomes thus altering the translation of specific mRNAs encoding oncogenic proteins such as IGF1R or CMYC [12, 15, 19]. *FBL* might thus represent a strong biomarker of ribosome biogenesis in cancer, in particular in breast cancers.

Although major advances have been made over the past 15 years, breast cancer still remains the most frequent cancer in women worldwide with about 2 million patients diagnosed in 2018 (*Globocan 2018, OMS*). Breast cancer-related death is intimately linked to the nature of the tumor since this heterogeneous disease encompasses several subtypes with distinct phenotypes, responses to therapy and thus clinical outcomes [20]. The strategy used for breast cancer patient management relies on the identification at diagnosis of breast cancer subtypes and characteristics to provide a therapeutic treatment specifically adapted to the tumor. Nevertheless 15–20% of breast cancer patients are still dying from their disease. One important issue for clinicians remains the identification of prognostic and predictive markers, including at early stage of the disease [21]. Here, we determine whether FBL is a marker of patient outcome at early stages of breast cancer that might reflect ribosome biogenesis activity.

## Methods

### Human breast tumors and healthy donors samples

A total of 3,275 primary breast tumors non-metastatic at diagnosis, encompassing six cohorts of breast cancer patients from four different institutions were analyzed: Tayside Tissue Bank of Dundee (TTBD, Dundee, Scotland, UK), The Cancer Genome Atlas (TCGA, NIH, USA) [22], BB-0033–00050 CRB Centre Léon Bérard (CLB, Lyon, France) and Institut Gustave Roussy (IGR, Villejuif, France) (Supplementary Table S1). A seventh series composed of 11 mammary tissues derived from healthy donors and issued from reduction mammoplasties was provided by the Institut Curie (Paris, France) [23]. Detailed information is available in the Supplementary Material and Methods.

### Gene expression analysis

Gene expression was quantified by medium-throughput real-time quantitative PCR using the HD Biomark system (Fluidigm) as in [24]. Relative fold-changes were calculated using the 2-ΔΔCT method. Using RNA-seq data derived from the TCGA series (RNA Seq V2 RSEM), transcriptomic analyses were performed using k-means clustering approach.

### FBL immunohistochemical staining

FBL immunohistochemistry (IHC) was performed using two different lots of FBL antibody for CLB-1 (ab5821 lot GR1979-001, Abcam) and IGR-1 series (ab5821 lot GR253838-1, Abcam). FBL staining corresponds to a nucleolar staining. Breast tumors were classified according to FBL immunostaining organization, i.e., the number of FBL dots per nucleus. Four different types of FBL immunostaining categories were detected: “single” (1 dot per cell); “multiple” (> 1 dot per cell); “heterogeneous” (mix of “single” and “multiple”); and finally “no detection”.

### Statistical analysis

Descriptive statistics were used to summarize the initial characteristics of patients. Survival curves with associated log-rank tests were generated using the Kaplan Meier method for overall survival (OS: from diagnosis to death), invasive disease-free survival (iDFS: from diagnosis to either locoregional relapse, metastasis detection, new breast cancer or death), distant disease-free survival (dDFS: from diagnosis to occurrence of metastases or death) and disease-free survival (DFS: from diagnosis to either relapse or death from all causes if no relapse had been observed). Univariate and multivariate Cox proportional hazards models were used to investigate confounding factors predictive of survivals. Statistical analyses were performed using either SAS v9.4 (SAS Institute), R v3.5.1 (package survival) or GraphPad Prism v7.0a (GraphPad Software, Inc) software.

## Results

### Lowest levels of FBL mRNA are associated with poor patient outcome at early stage of breast cancer

To decipher whether ribosome biogenesis factors, and in particular the rRNA methyltransferase *FBL*, could be exploited as a novel biomarker to identify breast cancer patients with the poorest outcome at an early stage diagnosis, we first analyzed the association between *FBL* mRNA expression and overall survival (OS) or disease-free survival (DFS) in a series of 216 breast tumors (TTBD series, Supplementary Table S1). We validated that the TTBD series displayed characteristics of a classical breast cancer population (data not shown).

**Fig. 1:**
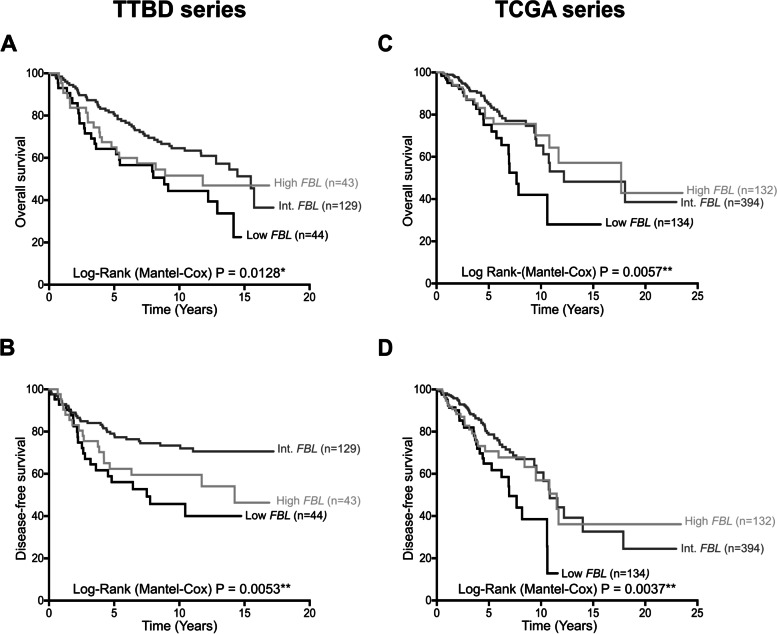
Association between *FBL* mRNA levels and survivals in two independent breast cancer series. Using the cut-offs identified in the Supplementary figure S2 for *FBL* mRNA expression levels, association between *FBL* mRNA levels and OS (**A**, **C**) and DFS (**B**, **D**) was determined using Kaplan–Meier analyses in the TTBD series (*n* = 216; **A**-**B**) and TCGA series (*n* = 661; **C**-**D**). An association between *FBL* mRNA expression and OS and DFS was observed in the two independent breast cancer series. Patients bearing breast tumors expressing either “low” or “high” *FBL* mRNA levels exhibited worse survival compared to tumors expressing “int.” *FBL* mRNA levels. Int.: Intermediate. *: *P* < 0.05; **:*P* < 0.01

Using the quartile and tercile distribution of *FBL* mRNA levels as initial cut-off values, we unexpectedly observed that three groups of *FBL*-related breast cancer patients exhibited different OS (quartile: *P* = 0.0725, tercile: *P* = 0.0745, Supplementary figure S1A-C). To avoid inclusion of borderline “intermediate” *FBL*-expressing tumors in both “low” and “high” *FBL* groups, more stringent *FBL* cut-off values were then refined to produce “low” [0–20%] (≥ 0 and ≤ 20%), “intermediate” ]20–80%] (> 20 and ≤ 80%) and “high” ]80–100%] (> 80 and ≤ 100%) *FBL* mRNA-related groups. Based on this *FBL*-related stratification, Kaplan–Meier curves revealed that patients harboring breast tumors expressing different *FBL* mRNA levels displayed distinct OS and DFS (*P* = 0.0128 and 0.0053, respectively, Fig. 1A-B). Univariate Cox regression analyses support this observation (Table 1, FBL set 1). Moreover, these data show that “low” and “high” FBL marker were associated with a hazard ratio (HR) > 1 when using the “intermediate” marker as a reference (OS: HR high: 1.40, CI95%: [0.84–2.34], HR low: 2.01, CI95%: [1.25–3.23], *P* = 0.0150; DFS: HR high: 1.51, CI95%: [0.93–2.45], HR low: 2.06, CI95%: [1.30–3.27], *P* = 0.0065), suggesting that patients bearing tumors with either “low” or “high” *FBL* mRNA levels displayed poor OS and DFS than the ones bearing tumours with “intermediate” *FBL* mRNA levels.

**Table 1:** Association between rRNA 2’-O-Me maturation complex factors and overall survival or disease-free survival using univariate Cox regression analyses in the TTBD series

	**Overall survival**			**Disease-free survival**		
**Variables**	**HR**	**CI95%**	***P*-value**	**HR**	**CI95%**	***P*-value**
***FBL* (set 1)**						
Intermediate	1.00		0.0150*	1.00		0.0065**
Low	2.01	[1.25–3.23]		2.06	[1.30–3.27]	
High	1.40	[0.84–2.34]		1.51	[0.93–2.45]	
***FBL* (set 2)**						
Intermediate	1.00		0.0615	1.00		0.0815
Low	1.69	[1.04–2.75]		1.58	[0.99–2.53]	
High	1.53	[0.92–2.54]		1.52	[0.94–2.46]	
***NHP2L1***						
Intermediate	1.00		0.0763	1.00		0.0538
Low	1.67	[1.05–2.66]		1.74	[1.11–2.72]	
High	1.05	[0.63–1.75]		1.20	[0.74–1.93]	
***NOP56***						
Intermediate	1.00			1.00		0.0604
Low	1.52	[0.93–2.50]	0.1165	1.46	[0.90–2.36]	
High	1.54	[0.96–2.46]		1.67	[1.07–2.61]	
***NOP58***						
High	1.00		0.0031**	1.00		0.0078**
Low	2.01	[1.27–3.20]		1.84	[1.17–2.88]	

*HR* Hazard Ratio, *CI95%* 95% of Confidence Interval

A similar observation between *FBL* mRNA expression levels and OS/DFS was made using a second set of *FBL* primers (OS: *P* = 0.0577; DFS: *P* = 0.0774; Supplementary figure S1D-E). In a second independent series of 661 breast samples, breast tumors expressing low *FBL* mRNA levels also displayed poor survivals (TCGA series, OS *P* = 0.0057, DFS *P* = 0.0037; Fig. 1C-D). Association between poor OS/DFS and high *FBL* mRNA levels was observed for the first 5 years. To reinforce these data, we performed complementary statistical analyses. Using *FBL* data derived from the two sets of primers in the TTBD series, univariate Cox regression analyses showed an association between *FBL* mRNA levels, in particular between “low” *FBL*, and poor OS (FBL set 2: HR high: 1.53, CI95%: [0.92–2.54], HR low: 1.69, CI95%: [1.04–2.75], *P* = 0.0615) and DFS (FBL set 2: HR high: 1.52, CI95%: [0.94–2.46], HR low: 1.58, CI95%: [0.99–2.53], *P* = 0.0815) (Table 1).

To further characterize the three tumor groups, and particularly to determine how the levels of *FBL* mRNA from tumor cells diverged from those of healthy cells, we compared *FBL* mRNA levels quantified in the TTBD series to those of 11 mastectomy samples from healthy donors (Supplementary figure S1F). Compared to healthy tissues, a significant decrease in *FBL* mRNA levels was observed in “low” *FBL* expressing tumors (*P* < 0.0001), suggesting that these tumors exhibit *FBL* underexpression. A significant gradual increase in *FBL* mRNA levels was observed in the “intermediate” and “high” *FBL* expressing tumors (*P* < 0.0001), suggesting that the “high” *FBL* expressing tumors displayed overexpressed *FBL* mRNA levels compared to normal tissues. Altogether, these data suggest that overexpression, but also and mainly underexpression, of *FBL* is associated with poor patient prognosis at an early stage of breast cancer.

### FBL is an independent marker of poor patient outcome in breast cancer at the mRNA level

To determine whether *FBL* is an independent marker of breast cancer outcome, we first performed univariate Cox regression analyses for *NHP2L1*, *NOP56* and *NOP58*, the three factors associated with *FBL* in the rRNA 2’-O-Me maturation complex (C/D box snoRNP complex) (Table 1) [12]. An association between *NHP2L1* or *NOP56* mRNA levels and OS or DFS was observed (*NHP2L1*: OS *P* = 0.0763 and DFS *P* = 0.0538, respectively; *NOP56*: OS *P* = 0.1165 and DFS *P* = 0.0604). However, a stronger and significant association was observed using a univariate Cox model between *NOP58* mRNA levels and OS (*P* = 0.0031) and DFS (*P* = 0.0078). A strong correlation between *FBL* and *NOP58* mRNA levels was observed in the TTBD series (*r* = 0.68, *P* < 0.0001, data not shown). Multivariate Cox regression model was performed to decipher whether both *FBL* and *NOP58* had each an independent effect on OS and/or DFS even after adjustment against gold standard prognostic factors, including tumor size, lymph node invasion status and breast cancer subtype (Tables 2 and 3). These models revealed that *FBL*, but not *NOP58*, remained associated with OS (Low *FBL*: HR: 2.35, CI95%: [1.41–3.92]; High *FBL*: HR: 1.27, CI95%: [0.72–2.24]; *P* = 0.0042) and DFS (Low *FBL*: HR: 2.02, CI95%: [1.21–3.39]; High *FBL*: HR: 1.56, CI95%: [0.94–2.56]; *P* = 0.0149). Therefore, *FBL* was identified as the only gene among the rRNA 2’-O-Me maturation complex of prognostic value in breast cancer.

**Table 2:** Multivariate Cox regression analyses for overall survival and disease-free survival using significant univariate variables in the TTBD series (Step with NOP58 that was removed from the model)

	**Overall survival**			**Disease-free survival**		
**Variables**	**HR**	**CI95%**	***P*-value**	**HR**	**CI95%**	***P*-value**
***FBL***						
Intermediate	1.00		0.0230*	1.00		0.0149*
Low	2.16	[1.24–3.74]		2.02	[1.21–3.39]	
High	1.32	[0.74–2.34]		1.56	[0.94–2.56]	
***NOP58***						
Low	1.00		0.4034	1.00		0.3036
High	1.27	[0.73–2.22]		1.32	[0.78–2.24]	
**Tumor size**						
< 30 mm	1.00		< 0.0001***	1.00		0.0004**
≥ 30 mm	2.70	[1.71–4.23]		2.07	[1.38–3.09]	
**Lymph node invasion status**						
*N* = 0	1.00		0.0386*	1.00		0.0180*
N ≥ 1	1.63	[1.02–2.60]		1.64	[1.09–2.47]	
**Breast cancer subtypes**						
ER + PR ± HER2-	1.00		0.0616	N/A		N/A
ER ± PR ± HER2 +	1.05	[0.62–1.76]				
ER- PR- HER2-	1.88	[1.08–3.26]				

*HR* Hazard Ratio, *CI95%* 95% of Confidence Interval

**Table 3:** Multivariate Cox regression analyses for overall survival and disease-free survival using significant univariate variables in the TTBD series (final multivariate model)

	**Overall survival**			**Disease-free survival**		
**Variables**	**HR**	**CI95%**	***P*-value**	**HR**	**CI95%**	***P*-value**
***FBL***						
Intermediate	1.00		0.0042*	1.00		0.0149*
Low	2.35	[1.41–3.92]		2.02	[1.21–3.39]	
High	1.27	[0.72–2.24]		1.56	[0.94–2.56]	
***NOP58***						
Low			*removed*			*removed*
High						
**Tumor size**						
< 30 mm	1.00		< 0.0001***	1.00		0.0002**
≥ 30 mm	2.77	[1.77–4.33]		2.14	[1.44–3.18]	
**Lymph node invasion status**						
*N* = 0	1.00		0.0443*	1.00		0.0174*
N ≥ 1	1.61	[1.01–2.55]		1.64	[1.09–2.48]	
**Breast cancer subtypes**						
ER + PR ± HER2-	1.00		0.0463*	N/A		N/A
ER ± PR ± HER2 +	1.06	[0.63–1.79]				
ER- PR- HER2-	1.94	[1.12–3.35]				

*HR* Hazard Ratio, *CI95%* 95% of Confidence Interval, *N/A* Not include in the model, *removed* NOP58 was included in the model but removed in step (a) of a backward step selection

An independent association between *FBL* and patient survivals after adjustment against gold standard prognostic factors was also obtained using the second set of *FBL* primers in the TTBD series for OS (Low *FBL*: HR: 2.10, CI95%: [1.27–3.47]; High *FBL*: HR: 1.82, CI95%: [1.07–3.11]; *P* = 0.0066) and DFS (Low *FBL*: HR: 1.83, CI95%: [1.13–2.96]; High *FBL*: HR: 1.69, CI95%: [1.02–2.80]; *P* = 0.0215) (data not shown). Interestingly, *FBL* mRNA levels significantly discriminated patients with different outcomes exhibiting either small tumor size or no invaded lymph node at early stage diagnosis (*P* < 0.0001 and 0.0073, respectively) (Supplementary figure S2A-D). Indeed, the combination of *FBL* mRNA level and tumor size highlighted three categories of breast cancer patients: patients with the best OS and DFS that carry small tumors expressing intermediate *FBL* mRNA levels; patients with the poorest OS and DFS that carry large tumors expressing low *FBL* mRNA levels; and patients with intermediate survival exhibiting other combinations. We next observed that “low” and “high” *FBL*-related breast cancer groups seemed to exhibit the same pattern of OS and DFS in specific breast cancer subtypes, including in ER + PR ± HER2- (corresponding to luminal subtype, *P* = 0.2485 and 0.2358, respectively) and in ER- PR- HER2- (corresponding to triple negative subtype, *P* = 0.0441 and 0.0323, respectively) (data not shown). This observation was not noticed in ER ± PR ± HER2 + tumors (corresponding to HER2-amplified subtype, *P* = 0.3868 and 0.1370, respectively). Overall, these data indicate that the *FBL* mRNA level is an independent marker of poor patient outcome at an early stage of breast cancer.

A validation series composed of 198 primary breast tumors was analyzed to sustain these observations (IGR-2 series, Supplementary Table S1). Since few events occurred in the IGR-2 series, we focused on the association of *FBL* mRNA levels with distant disease-free survival (dDFS) and we used the tercile as cut-off values, which were different and less stringent than the ones used for the TTBD and TCGA cohorts (Supplementary figure S2E). Kaplan–Meier curves (*P* = 0.032) and univariate Cox regression analyses (Low *FBL*: HR: 4.04, CI95%: [1.32–12.40], *P* = 0.0150; High *FBL*: HR: 3.14, CI95%: [0.98–10.02], *P* = 0.0540) showed that patients carrying tumors expressing either “low” or “high” *FBL* mRNA levels exhibited a significantly poorer dDFS than patients with tumors expressing “intermediate” *FBL* mRNA levels. In addition, multivariate Cox regression models built on current clinical markers revealed that the *FBL* mRNA levels remained a significant marker of dDFS (Low *FBL*: HR: 3.89, CI95%: [1.07–14.11], *P* = 0.0390; High *FBL*: HR: 3.92, CI95%: [1.11–13.91], *P* = 0.0344) (Supplementary Table S2). Altogether these data identify *FBL* as an independent marker of patient outcome at an early stage of breast cancer.

### Lack of FBL protein detection is associated with poor patient outcome in breast cancer

**Fig. 2:**
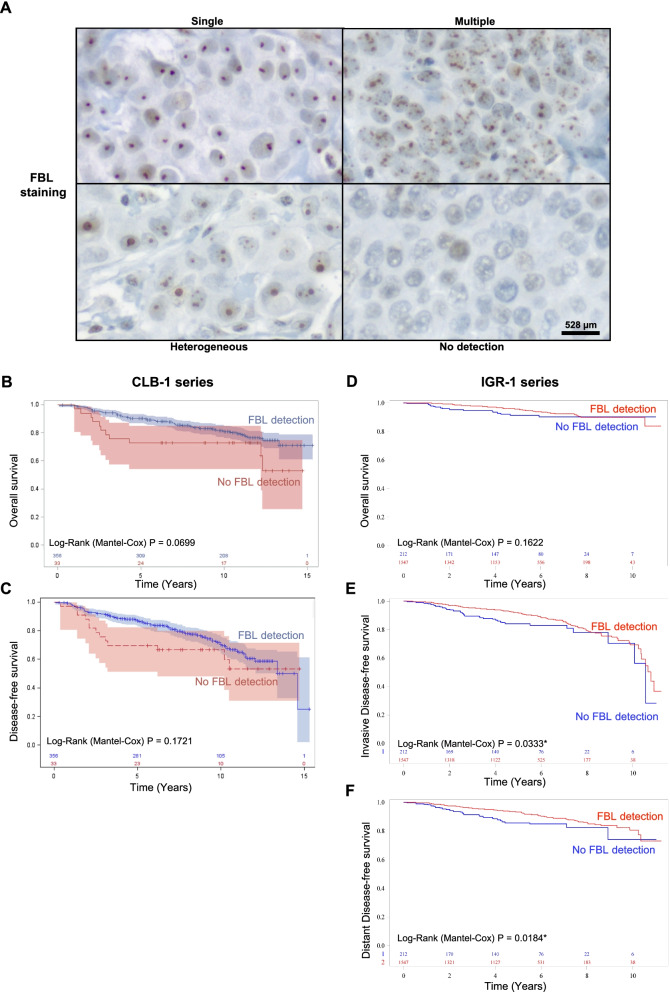
Association between FBL immunostaining and survivals in two independent breast cancer series. **A** In the two TMA series, FBL staining presented four different patterns based on the number of FBL dots per nucleus: “single”, “multiple”; “heterogeneous” and “no detection”. **B-F** Association between FBL immunostaining and OS (**B, D**), DFS (**C**), iDFS (**E**) and dDFS (**F**) was assessed using Kaplan–Meier analyses in CLB-1 (*n* = 389; **B-C**) and IGR-1 series (*n* = 1759; **D-F**). Patients harboring tumors with “no FBL detection” exhibited the poorest OS, DFS, iDFS and dDFS compared to patients with tumors that displayed FBL staining (i.e., tumors with “single” or “multiple” or “heterogeneous” FBL staining). *: *P* < 0.05 (**E–F**); scale bar: 528 µm

*FBL* expression at the protein level was analyzed by IHC in two TMAs: a test series of 389 primary breast tumors (CLB-1) and a validation series of 1,759 tumors (IGR-1) displaying characteristics of a classical breast cancer population (Supplementary Table S3). Western blotting analysis highlighted the strong specificity and efficacy of FBL antibody for FBL protein detection (Supplementary figure S3). Using IHC, FBL staining allowed a clear evaluation of the FBL intracellular distribution that corresponds to nucleolus location as expected [10]. However, due to the high and diverse number of nucleoli per tumor cells, evaluating difference in FBL expression level using IHC remains sensitive. Therefore, breast tumors were classified only on the basis of the different intracellular distributions of FBL in the tumor cells (Fig. 2A). FBL nucleolar staining exhibited either a single dot per cell (termed “single”), or multiple dots per cell (“multiple”) or a combination of single and multiple dots per cell (“heterogeneous”). We also identified samples in which the FBL signal was not detected (“no detection”). This latter group corresponded to 8.6% of tumor samples. A similar distribution of this “no detection” group was observed in the IGR-1 series, representing 12% of tumor samples (Supplementary figure S4A). These data supported the existence of breast tumors with undetectable FBL protein.

The association between the four breast cancer patient groups exhibiting different FBL staining and OS/DFS was analyzed using Kaplan–Meier curves (Supplementary figure S4B-C). No significant association was observed (*P* = 0.0988 and 0.4796, respectively). However, the patients bearing tumors with “no detection” displayed the poorest survival. We thus compared survival of patients with tumors exhibiting FBL staining or not (Fig. 2B-F). In the CLB-1 series, patients with a “no detection” tumor status tended to have a poorer OS and DFS than those of patients carrying tumors in which FBL staining was detectable (*P* = 0.0699 and 0.1721, respectively) (Fig. 2B-C). In the IGR-1 series, a significant association was observed using Kaplan–Meier and univariate Cox regression analyses between patients harboring “no detection” tumors and poor iDFS and dDFS (*P* = 0.0333 and 0.0184, respectively) (Fig. 2D-F and Table 4). These data suggest that “no detection” is a marker of poor patient outcome at early stages of breast cancer patients.

**Table 4:** Association between FBL immunostaining and overall survival or disease-free survival using univariate Cox regression analyses in the IGR-1 series

	**Survival**		
**Variables**	**HR**	**CI95%**	***P*-value**
***Overall survival (OS)***			
no detection	1.00		0.160
detection	0.68	[0.40–1.17]	
***Invasive Disease-free survival (iDFS)***			
no detection	1.00		0.034*
detection	0.67	[0.47–0.97]	
***Distant Disease-free survival (dDFS)***			
no detection	1.00		0.020*
detection	0.62	[0.41–0.93]	

*HR* Hazard Ratio, *CI95%* 95% of Confidence Interval

To ensure that the “no detection” status did not arise from lack of nucleoli preservation during sample processing, we analyzed nucleoli organization in the CLB-1 series using Hematoxylin/Phloxine Saffon (HPS) staining that allows visualization of subcellular compartments including nucleoli. We observed that all tumor samples displayed nucleoli, including the ones for which no detection of FBL staining was observed, suggesting that lack of detectable FBL was not related to their absence (Supplementary figure S4D). Using total RNA extracted from 41 randomly chosen samples issued from the CLB-1 series, we determined that tumors in which the FBL signal was not detected by IHC, expressed significantly lower *FBL* mRNA levels than the ones in which FBL staining was detected (*P* = 0.0063, Supplementary figure S4E). Overall, these data support the existence of primary breast cancer tumors lacking detection of FBL protein associated with low mRNA levels of *FBL* that are associated with poor patient outcome at early stages of breast cancer.

### Breast tumors expressing the highest and the lowest FBL mRNA levels exhibit distinct clinical and biological characteristics

To characterize the primary breast tumors expressing the highest and the lowest *FBL* mRNA levels, we first compared clinical characteristics of breast patients in the TTBD, CLB-1 and IGR-1 series. In the TTBD series, no significant difference was observed in the different *FBL* mRNA-related tumor groups (data not shown). Conversely, in the CLB-1 and IGR-1 series, tumors classified as “no detection” (i.e., low *FBL*) were associated with aggressive cancers exhibiting larger tumors, older patients (CLB-1 series, *P* = 0.033 and 0.012, respectively) and enrichment in triple negative breast tumors compared to tumors displaying FBL staining (IGR-1 series, *P* < 0.0001). These data suggest that breast tumors expressing the highest and the lowest levels of *FBL* exhibit different biological and clinical characteristics.

**Fig. 3:**
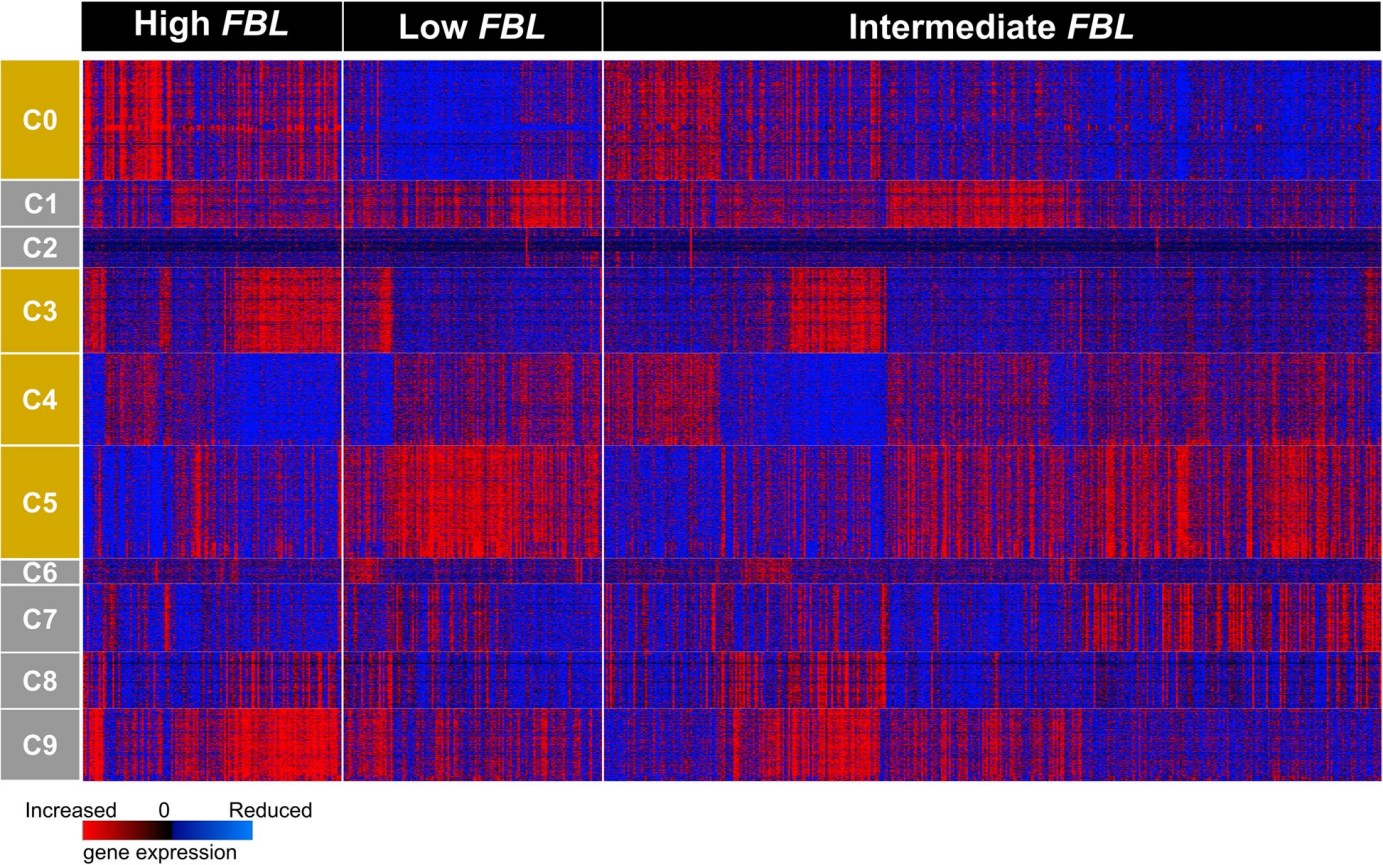
Differential gene expression profiles between the three groups of *FBL* mRNA levels-related groups. A heat-map was generated using transcriptomic data from the 661 primary breast tumors of TCGA series. “Low” and “high” *FBL* expressing tumors exhibited distinct gene expression profiles for some clusters (i.e., clusters 0, 3, 4 and 5). Blue: reduced expression level; red: increased expression level; orange: clusters with different signatures in “low” and “high” *FBL* expressing tumors. Gene ontology (GO) functional annotation clustering was performed using DAVID tools on the four clusters presenting difference in gene expression profiles between tumors expressing “low” or “high” *FBL* mRNA levels (Clusters 0, 3, 4 and 5). Enrichment of genes involved in translation was observed for the cluster 0, in glycosylation for the cluster 3 and in transcription for the clusters 4 and 5

Next, we analyzed the association between *FBL* mRNA-related groups and genomic or transcriptomic specificities of the tumors using TCGA series. At the genomic level, we first observed that breast tumors expressing low *FBL* mRNA levels had significantly lower number of copy alterations or mutation counts than breast tumors exhibiting high *FBL* mRNA levels, supporting that the two *FBL*-related tumor groups are different (Supplementary figure S5A-B). Using the transcriptome dataset of TCGA series and clustering approaches to compare gene expression profiles within the *FBL* mRNA-related groups of breast tumors, we observed that 4 out of the 10 gene-based clusters displayed different expression profiles between high and low *FBL*-related tumors (Fig. 3). These data indicate that the two *FBL*-related tumor groups have different biological properties. Interestingly the cluster 0 is enriched in genes encoding ribosomal proteins and proteins involved in translation as shown by gene ontology analyses. Comparison of median expression of the genes coding for the 80 human ribosomal proteins in the three *FBL*-related tumors showed a significant dose-dependent correlation between *FBL* mRNA expression levels (i.e., low, intermediate and high) and ribosomal protein expression levels, as expected due to the role of FBL in ribosome biogenesis [12] (Supplementary figure S5C). Overall, it appears that breast tumors overexpressing and underexpressing *FBL* exhibit different clinical characteristics and gene expression profiles, in particular regarding ribosome production and mRNA translation.

## Discussion

Our data identify the rRNA methyltransferase *FBL* as a strong ribosome biogenesis-related prognosis biomarker in non-metastatic breast cancer patients. A significant association between *FBL* expression and prognosis was obtained from the analyses of 6 independent breast cancer series representing a total of 3,275 samples. Despite differences between the test and validation series (three different European cancer centres with usage of different quantifiable events, statistical approaches, biological materials and mRNA quantification techniques), the data enabled us to draw similar conclusions, thus strengthening our findings. Interestingly, *FBL* remains an independent marker of poor prognosis at early stages of breast cancer even after adjustment against routinely used clinical gold standards. Such observations suggest that *FBL* could provide additional information compared to taking only clinical gold standards into account and could thus drastically improve patient stratification. For instance, in combination with tumor size, *FBL* expression led to the identification of patients with the poorest outcome although they harbored small tumors generally associated with a low risk factor in breast cancer patients. These breast cancer patients might either benefit from treatment usually used for large tumors or display poor outcome due to side-effects of their current treatment. Indeed, some patients who are at a low-risk of recurrence derive only a small benefit from adjuvant chemotherapies, which may be outweighed by long-term toxicities [21]. Identification of innovative biomarkers in such populations, like *FBL*, would allow the delivery of optimal treatments and de-escalation of therapies.

One of the most intriguing results of this study remains the identification of aggressive tumors expressing low levels of *FBL*, representing about 10% of all breast tumors. Although the definition of normal tissues regarding breast cancer disease is still a matter of controversy [25], we suggest that low *FBL* expressing tumors display reduced *FBL* expression levels compared to normal tissues. The existence of breast tumors underexpressing *FBL* identified at mRNA levels was supported by FBL immunostaining in TMAs: i) similar proportion of breast tumors exhibiting no FBL signal in two different breast tumour series; ii) preservation of nucleoli during sample processing; and iii) significant reduction of *FBL* mRNA levels in these tumors compared to FBL-stained tumors.

So far, all studies, including ours, demonstrated that *FBL* is overexpressed in breast tumors and is associated with poor patient outcome [15, 17, 18]. These data were accumulated taking into account molecular mechanisms available at the time of their publication. Indeed, we showed that *FBL* overexpression altered rRNA 2’-O-Me profiles thus affecting intrinsic activity of ribosomes and translational efficacy of some oncogenic mRNAs such as IGF1R or CMYC [15]. Moreover, *FBL* overexpressing breast cancer cell lines exhibited increased cell proliferation and resistance to chemotherapy, reinforcing the association between *FBL* overexpression and poor survivals [15]. Finally, the fact that *FBL* is an essential gene, the homozygous depletion of which is lethal [26–28], has so far prevented to formulate hypotheses regarding the putative role of *FBL* reduction in tumorigenesis. However, by performing a non-hypothesis-driven study using large sample sets, we identified unexpected reduction of *FBL* found in about 10% of all breast tumors. Interestingly, underexpressing *FBL* tumors are characterized by a reduction in ribosomal proteins compared to tumors overexpressing *FBL*. Thus, *FBL* might directly reflect amount of ribosomes in tumors, as expected due to the pivotal role of FBL in ribosome biogenesis [12]. We have also recently demonstrated that reduced expression of nucleolin (*NCL*), a regulator of RNA polymerase I activity finely regulating ribosome production, is associated with poor prognosis in breast cancer, supporting a relationship between reduced ribosome biogenesis and cancer outcome [24]. It has to be noted that in anal squamous cell carcinoma, molecular classification based on proteomic profile distinguish two groups displaying either low or high amount of ribosome and translation related proteins [29]. These data support the notion that low amount of ribosomes might be a new feature of some particular tumors.

Two non-mutually exclusive hypotheses could be proposed regarding the contribution of *FBL* reduction in tumorigenesis. First, association of low *FBL* with poor prognosis might result from a decrease in ribosome biogenesis. Several hereditary diseases are indeed characterized by reduction of the number of ribosomes associated with increased cancer susceptibilities although the cellular and molecular mechanisms by which quantitative alteration of ribosomes contributes to neoplastic transformation remain a matter of debate [9, 30]. Decreased in ribosomal content might either induces p53 activation that should be bypass for cell survival thus resulting in the selective loss of p53 and acquisition of neoplastic transformation, or impairs translation of some specific mRNAs encoding oncogenes and tumor suppressors. Second, association of low *FBL* with poor prognosis might results from qualitative alterations of ribosomes. Indeed, we recently reported that reduction of *FBL* expression in HeLa cells alters rRNA 2’-O-Me profiles and thus translational regulation by ribosomes [19]. Although rRNA 2’-O-Me profiles have not been compared in the same cell lines in response to alterations of *FBL* expression, it appears that sites exhibiting variations in rRNA 2’-O-Me level in response to a reduction or increase in FBL expression were different [15, 19]. Thus, overexpression and underexpression of *FBL* might differentially affect ribosome translational activities, including the rate of translation speed, by differentially modulating rRNA 2’-O-Me profiles. Hence, both alterations can support the high proliferative rate of cancer cells and promote development of tumors with distinct characteristics. Our data demonstrating that *FBL*, but not the other components of the rRNA 2’O-Me maturation complex, is an independent marker of poor prognosis, support the important role of *FBL* in cancer. Such hypotheses regarding its related biological functions remain to be tested in the near future to sustain a dual role for *FBL* in tumorigenesis.

## Conclusions

*FBL* appears as a novel independent marker of poor patient outcome in breast cancer that belongs to the emerging field of ribosome in oncology. In contrast to AgNOR reflecting nucleoli sub-cellular compartments that are always present in cancer cells but with different shapes and numbers rendering histological reading difficult, *FBL* expression corresponds to a classical gene-based biomarker easily applicable in the clinic. Furthermore, based on the recent demonstration that targeting ribosome biogenesis primarily through DNA integrating molecules is a specific and efficient strategy to target cancer [3, 4], our discovery may pave the way for therapeutic opportunities by directly targeting FBL, in particular in high *FBL* expressing breast cancers. Finally, the identification of breast tumors expressing low levels of *FBL* suggests that reduced amount of ribosomes might be a novel molecular feature of a particular set of tumors.

## Supplementary Information

**Additional file 1:** **Supplementary Methods.** **Supplementary figure 1.** Association between FBL mRNA levels and patient survival. **Supplementary figure 2.** Improvement of breast cancer patient stratification derived from tumor size and lymph node invasion status using FBL mRNA expression. **Supplementary figure 3.** Validation of FBL antibodies used for FBL immunostaining. **Supplementary figure 4.** Association between FBL immunostaining and patient survival in CLB-1 and IGR-1 series. **Supplementary figure 5.** Characterization of the three tumor groups expressing different FBL mRNA levels. **Supplementary table 1**. Characteristics of patients from TTBD, IGR-2 and TCGA RNA breast cancer series. **Supplementary table 2.** Multivariate Cox regression analyses of FBL mRNA expression and gold standard prognostic factors with distant disease-free survival in IGR-2 series. **Supplementary table 3.** Characteristics of patients from the CLB-1 and IGR-1 breast cancer series.

## Data Availability

TCGA series corresponded to 818 samples obtained from TCGA Breast Invasive Carcinoma project of the public database cBioPortal (https://cbioportal.org).

## Abbreviations

AgNOR: Argyrophilic nucleolar organiser region
DFS: Disease-free survival
dDFS: Distant disease-free survival
ER: Estrogen receptor
FBL: Fibrillarin
HR: Hazard ratio
CI95%: Confidence interval 95%
iDFS: Invasive disease-free survival
OS: Overall survival
PR: Progesterone receptor
rRNA: Ribosomal RNA
