# Supplementary material for: Low level of Fibrillarin, a ribosome biogenesis factor, is a new independent marker of poor outcome in breast cancer
Source: BMC Cancer. 2022 May 11;22:526. doi: 10.1186/s12885-022-09552-x (PMC9092774; doi:10.1186/s12885-022-09552-x)
Supplement: Supplementary file 1 — Additional file 1: Supplementary Methods. Supplementary figure 1. Association between FBL mRNA levels and patient survival. Supplementary figure 2. Improvement of breast cancer patient stratification derived from tumor size and lymph node invasion status using FBL mRNA expression. Supplementary figure 3. Validation of FBL antibodies used for FBL immunostaining. Supplementary figure 4. Association between FBL immunostaining and patient survival in CLB-1 and IGR-1 series. Supplementary figure 5. Characterization of the three tumor groups expressing different FBL mRNA levels. Supplementary table 1. Characteristics of patients from TTBD, IGR-2 and TCGA RNA breast cancer series. Supplementary table 2. Multivariate Cox regression analyses of FBL mRNA expression and gold standard prognostic factors with distant disease-free survival in IGR-2 series. Supplementary table 3. Characteristics of patients from the CLB-1 and IGR-1 breast cancer series. [file 12885_2022_9552_MOESM1_ESM.zip › 2022-02-08_NGUYEN_SuppData_clean_R1.docx]

*Supplementary data to*

**Low level of Fibrillarin, a ribosome biogenesis factor, is a new independent marker of poor outcome in breast cancer**

Flora Nguyen Van Long^1,2,3^, Audrey Lardy-Cleaud^4^, Dimitri Carène^5,6^, Caroline Rossoni^6^, Frédéric Catez^1,2,3^, Paul Rollet^1,2,3^, Nathalie Pion^1,2,3^, Déborah Monchiet^1,2,3^, Agathe Dolbeau^1,2,3^, Marjorie Martin^1,2,3^, Valentin Simioni^1,2,3^, Susan Bray^7^, Doris Le Beherec^8^, Fernanda Mosele^5^, Ibrahim Bouakka^5^, Amélie Colombe-Vermorel^9^, Laetitia Odeyer^9^, Alexandra Diot^10^, Lee B. Jordan^11^, Alastair M. Thompson^10,12^, Françoise Jamen^13,14^, Thierry Dubois^15^, Sylvie Chabaud^4^, Stefan Michiels^6^, Isabelle Treilleux^9^, Jean-Christophe Bourdon^10^, David Pérol^4^, Alain Puisieux^1,3^, Fabrice André^5^, Jean-Jacques Diaz^1,2,3,*^ and Virginie Marcel^1,2,3,*^

^1^ Université Claude Bernard Lyon 1, INSERM 1052, CNRS 5286, Léon Bérard Cancer Centre, Cancer Research Center of Lyon, Lyon, 69373 cedex 08, France

^2^ Institut Convergence PLAsCAN, Lyon, 69373 cedex 08, France

^3^ DevWeCan Labex Laboratory, Lyon, 69373 cedex 08, France

^4^ Biostatistics unit, Department of Clinical Research, Léon Bérard Cancer Centre, 28 rue Laennec 69008 Lyon, France

^5^ Predictive biomarkers and novel therapeutic strategies Group, Institut Gustave Roussy, University of Paris Sud, INSERM 981, Université Paris Saclay, 114 rue Edouard Vaillant, 94800, Villejuif, France

^6^ Department of Biostatistics and Epidemiology, Institut Gustave Roussy, 94800, Villejuif, France

^7^ Tayside Tissue Bank, Ninewells Hospital and Medical School, NHS Tayside, Dundee, Scotland, DD1 9SY, UK

^8^ Department Translational Research, Institut Gustave Roussy, 94800, Villejuif, France

^9^ Department of Translational Research and Innovation, Léon Bérard Cancer Centre, 28 rue Laennec 69008 Lyon, France

^10^ Division of Cancer Research, University of Dundee, Ninewells Hospital and Medical School, Dundee, Scotland DD1 9SY, United Kingdom

^11^ Department of Pathology, University of Dundee, Ninewells Hospital and Medical School, Dundee, Scotland DD1 9SY, United Kingdom

^12^ Olga Keith Wiess Chair of Surgery, Dan L. Duncan Breast Center, Division of Surgical Oncology, Baylor College of Medicine, Houston, TX 77030, USA

^13^ Université Paris-Saclay Institute of Neuroscience, CNRS UMR9197, Gif-sur-Yvette, France

^14^ Université Paris-Saclay, CIAMS, 91405 Orsay Cedex, France

^15^ Breast Cancer Biology Group, Translational Research Department, Institut Curie-PSL Research University, 26 rue d’Ulm, 75005 Paris, France

^*^ Co-senior and corresponding authors:

Virginie Marcel: [virginie.marcel@lyon.unicancer.fr](mailto:virginie.marcel@lyon.unicancer.fr)

Jean-Jacques Diaz: [jean-jacques.diaz@lyon.unicancer.fr](mailto:jean-jacques.diaz@lyon.unicancer.fr)

Centre de Recherche en Cancérologie de Lyon, Cheney A, 28 rue Laennec, 69373 Lyon cedex 08

**Contents of Supplementary data**

Supplementary methods: pages 3 – 9

Supplementary figure legends: pages 10 – 12

Supplementary tables: pages 13 – 17

Supplementary references: page 18

**Supplementary Methods**

***Human breast tumors and healthy donor samples***

Six cohorts of breast cancer patients were analyzed from four different institutions: Tayside Tissue Bank of Dundee (www.tissuebank.dundee.ac.uk) (TTBD, Dundee, Scotland, UK), The Cancer Genome Atlas (www.cancer.gov) (TCGA, NIH, USA) (1), BB-0033-00050 CRB Centre Léon Bérard (CLB, Lyon, France) and Institut Gustave Roussy (IGR, Villejuif, France) (Supplementary Table S1). For consistency among the six cohorts, all series were composed of women with primary breast cancers, no family history, no neoadjuvant treatment and no distant metastasis detected at diagnosis. A seventh series composed of mammary tissues derived from healthy donors and issued from reduction mammoplasties was provided by the Institut Curie (Paris, France) (2).

TTBD series consisted of 216 primary breast tumor samples from the Tayside Tissue Bank of Dundee and received approval from the Local Research Ethics Committee of the Tayside Tissue Bank (REC Reference 07/S1402/90) (3,4). Tumor grades were defined according to cancer cell proliferation and differentiation statuses, while tumor stages were determined based on the TNM staging system (T: tumor size; N: number of invaded lymph nodes; and M: presence of metastases) (Supplementary Table S1). Tumors were categorized as “small” when tumor size was < 30 mm whereas “large” tumors exceeded 30 mm. Lymph node invasion status was considered “positive” when at least one invaded lymph node was detected at diagnosis. HER2 receptor status and hormonal receptor statuses (ER: estrogen receptor; and PR: progesterone receptor) were determined by immunohistochemistry (IHC) on paraffin-embedded tumors. Tumor breast cancer subtypes were assessed based on hormonal and HER2 receptor statuses. Tumors presenting a ER+ PR+/- HER2- status were considered to be equivalent to the luminal subtype, ER+/- PR+/- HER2+ tumors were equivalent to the HER-enriched subtype and ER- PR- HER2- tumors were equivalent to the triple-negative breast cancer subtype (TNBC). Total RNA were extracted from 10 mg of tumor tissue as described in a previous study (3).

TCGA series corresponded to 818 samples obtained from TCGA Breast Invasive Carcinoma project of the public database cBioPortal (cbioportal.org) (1). Normalized z-Score mRNA expression (RNA Seq V2 RSEM) was downloaded. Of note, 157 patients that were not women, having a family history or metastasis at diagnosis, were excluded from the study resulting of a total of 661 patients analyzed for this cohort (Supplementary Table S1). For consistency between the different cohorts, breast cancer subtypes were assigned using ER, PR and HER2 statuses, and lymph node invasion status was determined using TTBD series criteria.

CLB-1 series available from the Centre Léon Bérard encompassed 389 formalin-fixed and paraffin-embedded (FFPE) primary tumors that were assembled in triplicate into tissue microarrays (TMA) blocks (Supplementary Table S3). Tumor grade was assessed using the Scarff-Bloom-Richardon (SBR) grading system, which is based on three morphological features, the cancer cell mitotic activity, nuclear polymorphism and differentiation. Similar characteristics as TTBD series were used to define tumor size and lymph node invasion status. Hormonal receptor statuses were determined using IHC and HER2 receptor by both IHC and fluorescence *in situ* hybridization assay. Breast cancer subtypes were determined using hormonal receptor statuses (HR, composed of ER and PR), HER2 status, SBR grade and mitotic index: luminal A subtype corresponded to HR+ HER2- tumors with SBR grade 1 or SBR grade 2 with low mitotic index; luminal B to HR+ HER2- tumors with SBR grade 2 with high mitotic index or SBR grade 3 or HR+ HER2+ tumors; HER2-enriched subtype to HR- HER2+ tumors; and TNBC subtype to HR- HER2- tumors. The CLB-2 series corresponded to total RNA extracted from 41 available frozen biopsies derived from tumors of the CLB-1 series to compare *FBL* mRNA expression and FBL IHC staining. Total RNA extraction was performed by the CRB Centre Léon Bérard. Explanatory variables were extracted from the Centre Léon Bérard institutional breast cancer database, including those of all patients who underwent initial surgery performed at the institution since 1996. The database has been declared to the French authorities (CNIL, breast cancer database n° 447918).

The IGR-1 series comprised 1759 primary tumors from the Institut Gustave Roussy, which were either fixed in formalin or in alcohol-formalin-acetic acid (AFA), and were included in TMA (Supplementary Table S3). Lymph node invasion status was defined using the same criteria as TTBD series. Breast cancer subtypes were determined using ER and HER2 receptor statuses, independently of PR, ER+ HER2- corresponding to luminal tumors, ER- HER2+ to HER2-amplified tumors and ER- HER2- to TNBC. The IGR-2 series corresponded to total RNA extracted from 198 available frozen biopsies issued from the Institut Gustave Roussy (Supplementary Table S1). Biopsies were ground in TRIzol Reagent (ThermoFisher Scientific) using 2 cycles at 6,000 rpm for 30 s with the Precellys Evolution homogenizer (Bertin instruments). For complete tissue homogenization another cycle of 10,000 rpm for 30 s was added for the larger biopsies. To maintain the temperature at 4°C during biopsy grinding, a Cryolys instrument (Bertin instrument) was associated to the Precellys device. Total RNA extraction was performed following the manufacturer’s instructions (ThermoFisher Scientific). 30 μg of total RNA were purified using RNeasy Mini Kit and DNAse treatment was included (Qiagen). Purified-total RNA quality was assessed by loading 250 ng of RNA on a 1% Tris-acetate-EDTA agarose gel (Euromedex) with 0.5 ug/mL Ethidium Bromide (Euromedex). Breast cancer subtypes were determined using HR and HER2 receptor statuses, HR+ HER2- corresponding to luminal tumors, HER2+ to HER2-amplified tumors and HR- HER2- to TNBC. Written consent was given by patients at the diagnosis to the inclusion of their biological material.

The Curie series was composed of 11 healthy samples issued from female mammary plastic surgery (2). Total RNA were extracted and obtained from the Biological Resource Center of the Institut Curie as previously described. The Bioethics Law No. 2004-800, the French National Institute of Cancer Ethic Charter and the ethics committee of the Institut Curie approved the use of this cohort.

**RT-qPCR**

200 ng of total RNAs were reverse transcribed using M-MLV kit (Invitrogen) for TTBD and the Curie series or using PrimeScript Reverse Transcriptase (Takara) for CLB-2 and IGR-2 series according to the manufacturer’s instructions (5).

Medium throughput RT-qPCR was performed using breast cancer series (5). cDNAs were pre-amplified during a multiplex PCR using primers of the different genes of interest and Pre-Amp Master Mix (Fluidigm) followed by an Exonuclease I treatment (New England BioLabs). cDNAs were then diluted at 1:5 in 1X Tris-EDTA buffer (Promega). Gene expression was quantified by medium throughput real-time quantitative PCR using the HD Biomark system (Fluidigm) coupled with Master Mix 2X EvaGreen (BioRad) and either the GE 48.48 Dynamic Array DNA Binding Dye Sample & Assay Loading reagent kit (Fluidigm) for TTBD series or the GE 96.96 Dynamic Array DNA Binding Dye Sample & Assay Loading reagent kit (Fluidigm) for the other RNA series. Relative fold-changes (FC) were calculated using the 2-ΔΔCT method by the Fluidigm Real-Time PCR Analysis software (v 3.1.3, Fluidigm) and normalized with Xpress Ref Universal Total RNA (Qiagen) as sample control (5,6). For TTBD and the Curie series, *GAPDH* was used as housekeeping gene while for CLB-2 and IGR-2 series, the median Ct value of five housekeeping genes was used (*β-actin, GAPDH, HPRT1, PGK1 and PPIA*). For each sample, two independent RT were performed and qPCR on each cDNA were performed in triplicate, six values being thus used to calculate FC.

Low throughput RT-qPCR was performed using a Light Cycler 480 II instrument (Roche) coupled with the Light Cycler 480 SYBR Green I Master Mix (Roche) to calibrate primers or quantify *FBL* expression levels in relatively few samples.

**FBL immunohistochemistry**

For the CLB-1 series, immunohistochemical staining was performed on 4-µm thick sections of formalin-fixed, paraffin-embedded tumors. The different steps from de-paraffinization and epitope retrieval to immunodetection were performed using the Discovery XT (Roche-Ventana) automate. Slides were counterstained using a Hematoxylin Phloxine Saffron solution (Roche) and Bluing (Roche). Slide mounting was done using the Tissue-Tef film (SAKURA Finetek) instrument. FBL antibody (ab5821 lot GR1979-001, Abcam) diluted at 1:400 was incubated for 1 h before detection using the Omnimap anti-rabbit conjugated to Horseradish Peroxydase kit (Roche) with ChromoMap 3,3–diaminobenzidine (DAB) reagent (Roche).

For the IGR-1 series, two types of fixation were used. 559 breast tumors were fixed in formalin and 1,200 tumors by alcohol-formalin-acetic acid (AFA). A similar automated protocol was carried out as for FBL immunostaining (ab5821 lot GR253838-1, Abcam).

FBL immunostaining was visualized as brown dots in the nucleolus. Breast tumors were classified according to nucleolar FBL immunostaining, meaning the number of FBL dots per nucleus. Four different types of FBL immunostaining were detected: “single” corresponding to the presence of one FBL dot per nucleus; “multiple” to the presence of several FBL dots per nucleus; “heterogeneous” to the presence of cells exhibiting either single or multiple FBL dots per nucleus; and finally “no detection” to the absence of detectable FBL immunostaining. FBL immunostaining were performed by two independent experimenters.

Image acquisition was done on a Zeiss AxioImager 2 microscope. White balance and JPG export were performed using the ZEN Blue software (v 2.5.75.2).

**Cell culture and siRNA transfection**

HeLa cells were maintained in Minimum Essential Medium (MEM) (Life Technologies) supplemented with 10% foetal bovine serum (Life Technologies), 1% penicillin-streptomycin (Life Technologies), 1% non-essential amino acids (Life Technologies) and 1% L-glutamine (Life Technologies) at 37°C with 5% CO_2_. A mix of three siRNA duplexes were used to silence FBL: 5'- UUU CCU CGA CAA AUG AAG AC – 3'; 5'- AAA AUC ACA AAG UGU CCU CCA -3'; and 5'- UCU CUC GCA AUC CUG ACA G -3' (Eurogentec) as previously described (7). A negative siRNA duplex control (Eurogentec) was also used as negative control of transfection. Briefly, cells were transfected for 96 h using X-tremeGene siRNA Transfection Reagent (Roche) according to the manufacturer’s protocol.

**Western blot analysis**

20 μg of total protein lysates prepared using 2X Laemmli buffer were loaded on a 12.5% polyacrylamide gel (Bio-Rad) and transferred to a nitrocellulose membrane (GE healthcare Life Sciences) using the Trans Blot Turbo Transfer System (Bio-Rad). Membranes were blocked with 5% non-fat milk in Tris Buffered Saline with Tween 20 (TBST) buffer. Antibodies were incubated for 1 h at room temperature in 2.5% non-fat milk in TBST (FBL: ab5821 lots GR1979-001 and GR253838-1, Abcam, 1:1,000; β−Actin as loading control: A5441, Sigma, 1:5,000). Proteins were detected using an anti-rabbit and anti-mouse peroxidase conjugated antibody (Cell signalling) at 1:10,000 in TBST and Clarity Western ECL Substrate (Bio-Rad). The chemiluminescence signal was detected with the ChemiDoc XRS+ system (Bio-Rad) and analyzed using the Image Lab software (v 5.1, Bio-Rad).

**Transcriptomic analysis**

Based on RNA-seq data from the TCGA series (RNA Seq V2 RSEM) (1), bioinformatics analyses were performed as previously described (5). Two-rounds of clustering analyses were performed using the k-means clustering approach. A first clustering was performed independently in the three groups of *FBL* expression to identify tumors within the three *FBL*-based groups that exhibited similar expression profiles. A second clustering was performed on the three groups by conserving the previously identified tumor clustering to identify putative differences in gene expression between the three *FBL*-based groups of breast tumors. Finally, gene ontology was performed using DAVID tools (functional annotation clustering) (8). Bioinformatics analyses were performed using R.

**Statistical analysis**

Descriptive statistics were used to summarize initial patient characteristics. Comparison of *FBL* expression levels between the *FBL* expression-based groups of breast tumors with healthy donors was performed using a Chi-2 or Fisher’s exact test for categorical data or non-parametric Kruskal-Wallis’ and Wilcoxon’s tests for continuous data.

Cut-offs of gene expression levels were determined using quartile/tercile-based overall survival in the TTBD series. Regarding *FBL*, three groups of expression levels were then refined based on *FBL* mRNA level distribution: tumors expressing “low” *FBL* expression levels that corresponds to [0-20%] of *FBL* expression levels (≥0 and ≤20%) (i.e., 20% of the tumours expressing the lowest *FBL* mRNA levels); “intermediate” *FBL* expression levels that corresponds to ]20-80%] of *FBL* expression levels (>20 and ≤80%); and “high” *FBL* expression levels that corresponds to ]80-100%] of *FBL* expression levels (>80 and ≤100%) (i.e., 20% of the tumours expressing the highest *FBL* mRNA levels). The same cut-off was applied to TCGA series. Cut-off based on the tercile distribution was applied on the IGR-2 series (low: [0-33%]; intermediate: ]33-66%]; and high: ]66-100%]).

Three endpoints defined according to DATECAN (Definition for the Assessment of Time-to-event Endpoints in CANcer trials) criteria were used (9). Overall survival (OS) corresponded to the timing from date of diagnosis to either death from all causes or last follow-up for censored patients. Disease-free survival (DFS) corresponded to the timing from date of diagnosis to either relapse, death from all causes (if no relapse had been observed) or last follow-up for censored patients. Invasive disease-free survival (iDFS) corresponded to the timing from data of diagnosis to either loco regional relapse, metastasis detection, new breast cancer, death from all causes or last follow-up for censored patients. Distant disease-free survival (dDFS) corresponded to the timing from date of diagnosis to the either metastasis detection, death or last follow-up for censored patients. Survival curves for OS, DFS, iDFS and dDFS with associated log-rank tests were generated using the Kaplan-Meier method. The survival median was estimated using the inverted Kaplan-Meier method.

A Cox proportional hazards model was used to verify the breast cancer series corresponded to standard breast cancer population and to investigate confounding factors predictive of OS and DFS. To investigate confounding factors, we performed multivariate models. Variables sufficiently informed (less than 10% missing value in univariate analyses or all relevant clinical information independently of univariate analyses, respectively for TTBD and IGR-2) and significant at a 5-10% level were included in a backward selection procedure to keep factors significant at a 5% level in the final multivariate model. For the multivariate model, P-values were given either as per variable or per modality.

All P-values corresponded to two-tailed P-values. A P-value < 0.05 was considered statistically significant. Statistical analyses and graphical representations were performed using either SAS v9.4 (SAS Institute), R v3.5.1 (package survival) or GraphPad Prism v7.0a software (GraphPad Software, Inc).

**Supplementary Figure Legends**

**Supplementary figure 1. Association between *FBL* mRNA levels and patient survival.** (**A**) In the TTBD series, patients were divided into groups according to *FBL* mRNA levels expressed in their tumors using either the quartile or the tercile distribution as cut-offs. (**B-C**) Association between *FBL* mRNA levels and OS using quartile (**B**) or tercile (**C**) distributions as cut-off values was assessed by Kaplan-Meier analyses. Patients who bore tumors expressing the highest (**B**: Q4; and **C**: T3) and the lowest (**B**: Q1; and **C**: T1) mRNA levels of *FBL* have poor OS compared to patients carrying tumors expressing intermediate levels of *FBL* (**B**: Q2-Q3; and **C**: T2). (**D-E**) Using the cut-offs identified in the TTBD series, association of *FBL* mRNA levels and OS (**D**) and DFS (**E**) was determined using Kaplan-Meier analyses on data from the second set of *FBL* primers in the TTBD series. A similar observation for association between *FBL* mRNA levels and OS and DFS was made using a second set of primers. (**F**) *FBL* mRNA levels were quantified in mastectomy tissues from healthy donors by medium throughput RT-qPCR and compared those of the three *FBL*-related tumor groups from the TTBD series (low *FBL*, intermediate *FBL* and high *FBL*). The “high” and “int.” tumors expressed significantly higher *FBL* mRNA levels compared to healthy donors, while the “low” tumors expressed significantly reduced *FBL* mRNA levels compared to healthy donors. Significance was assessed using the Log-Rank (Mantel-Cox) test, a Kruskal-Wallis test and the non-parametric Mann-Whitney-Wilcoxon test for two-by-two comparison. ***: P < 0.001; T: tercile; Q: quartile; Int.: intermediate.

**Supplementary figure 2. Improvement of breast cancer patient stratification derived from tumor size and lymph node invasion status using *FBL* mRNA expression.** (**A-D**) Association of tumor size (**A-B**) or lymph node invasion status (**C-D**) with OS (**A, C)** or DFS (**B, D**) was determined using Kaplan-Meier analyses in TTBD series. (**A-B**) Patients carrying large tumors (size ≥ 30 mm) and expressing “low” *FBL* mRNA levels exhibited the poorest survival, while patients with smallest tumors (size < 30mm) and expressing “int.” mRNA levels of *FBL* had the best survivals. (**C-D**) Patients harboring tumors with invaded lymph nodes (N ≥ 1) and expressing “low” mRNA levels of *FBL* had the poorest survivals, while patients with tumors in which no lymph node were invaded (N = 0) and expressing “int.” mRNA levels of *FBL* exhibited the best survivals. (**E**) Using the tercile as cut-off values, association between *FBL* mRNA levels and dDFS survival was assessed using Kaplan-Meier analyses in the IGR-2 series. A significant association between *FBL* mRNA levels and dDFS was observed. Significance was assessed using the Log-Rank (Mantel-Cox) test *: P ≤ 0.05; **: P < 0.01; ***: P < 0.001; N: number of invaded lymph nodes; S: small tumor size; L: large tumor size; Low: “low” *FBL* mRNA levels; Int.: “intermediate” *FBL* mRNA levels; High: “high” *FBL* mRNA levels.

**Supplementary figure 3. Validation of FBL antibodies used for FBL immunostaining**. (**A**) HeLa cells were transfected for 96 h with either a siRNA control (siCTRL) or a FBL siRNA (siFBL) before validating the decreased expression in *FBL* by siRNA at the mRNA level using RT-qPCR. Compared to siCTRL condition, a 5-fold decrease in *FBL* mRNA expression was observed in the siFBL condition. (**B-C**) Specificity and efficiency of FBL protein detection by the antibodies used for immunostaining was assessed by Western blot analysis in siCTRL and siFBL conditions. Two different FBL antibody lots were tested, one lot corresponding to the one used to stain the CLB-1 series (**B**) and the second lot corresponding to the one used to stain the IGR-1 series (**C**). For the two lots, only one band was observed even at 600 s exposure, corresponding to the predicted size of FBL protein and showing a decreased signal in siFBL compared to siCTRL condition. Full-length blots are presented in Original Data -1 file. Error bars represent a standard deviation of four independent experiments. ***: P< 0.001; kDa: kilo daltons; chemiluminescence signal: 7.1 s (corresponding to normal exposure for FBL protein detection) and 600 s (corresponding to overexposure).

**Supplementary figure 4. Association between FBL immunostaining and patient survival in CLB-1 and IGR-1 series.** (**A**) Distribution of the four groups showing different nucleolar FBL immunostaining (i.e., single, multiple, heterogeneous and no detection) were compared between the two TMA series, CLB-1 (n = 389) and IGR-1 (n = 1759). In the two independent series, the four groups exhibited almost similar proportions. (**B-C**) Association between FBL immunostaining and OS (**B**) and DFS (**C**) in the CLB-1 series was assessed using Kaplan-Meier analyses. Patients harboring tumors with “no detection” exhibited the poorest survivals compared to patients with tumors presenting the other types of FBL staining. Significance was assessed using the Log-Rank (Mantel-Cox) test. (**D**) Comparison of FBL immunostaining and Hematoxylin/Phloxine Saffron (HPS) staining used to identify cellular nucleoli was performed in the CLB-1 series. While no FBL signal was detected in the “no FBL detection” group, nucleoli were present in those tumors according to HPS staining. (**E**) *FBL* mRNA levels were quantified by RT-qPCR in 41 breast tumors from the CLB-2 series that displayed different nucleolar FBL immunostaining. Patients bearing tumors with “no FBL detection” expressed a significantly lower level of *FBL* mRNA than patients with tumors displaying other types of FBL staining. **: P < 0.01.

**Supplementary figure 5. Characterization of the three tumor groups expressing different *FBL* mRNA levels.** (**A-B**) Using the TCGA series (n = 661), comparison between the three tumor groups exhibiting different *FBL* mRNA levels (i.e., “low”, “intermediate”, “high”) and copy number alterations (**A**) or number of mutations (**B**) was performed. Tumors expressing “high” levels of *FBL* mRNA presented significantly higher copy number alterations and mutational counts than tumors expressing either “intermediate” or “low” *FBL* mRNA levels. (**C**) Median expression of mRNAs encoding the 80 human ribosomal proteins was compared in the three *FBL*-related groups of tumors in the TCGA series. A dose-dependent relationship was observed between *FBL* mRNA levels and the median expression of the 80 ribosomal proteins. ns: non significant; *: P < 0.05; **: P < 0.01; ***: P < 0.001, ****: P < 0.0001.

**Supplementary Tables**

**Supplementary table 1 – Characteristics of patients from TTBD, IGR-2 and TCGA RNA breast cancer series**

|  | **TTBD** | | | | | **IGR-2** | | | **TCGA** | | | | |
| --- | --- | --- | --- | --- | --- | --- | --- | --- | --- | --- | --- | --- | --- |
|  | | | (n = 216) | | | (n = 198) | | | (n = 661) | | | | |
|  | | |  | | |  | | |  | | | | |
| **Age (years)** | | | | | |  | | |  | | | | |
| Min | | | 28 |  | | 23 | | | 26 | | |  | |
| Max | | | 90 |  | | 93 | | | 90 | | |  | |
| Median | | | 59.0 | |  | 63.0 | |  | 58.0 | | | |  |
|  | | |  |  | |  |  | |  | |  | | |
| **Tumor size *** | | | | | |  | | |  | | | | |
| Missing data | | | 12 |  | | 0 | | | 1 | | |  | |
| Small | | | 117 | (57.4%) | | 66 | (33.3%) | | 180 | (27.3%) | | | |
| Large | | | 87 | (42.6%) | | 132 | (66.7%) | | 480 | (72.7%) | | | |
|  | | |  |  | |  |  | |  | |  | | |
| **Lymph node invasion status** | | | | | |  | | |  | | | | |
| Missing data | | | 0 |  | | 0 | | | 7 | | |  | |
| N = 0 | | | 98 | (45.4%) | | 122 | (61.6%) | | 334 | | (51.1%) | | |
| N ≥ 1 | | | 118 | (54.6%) | | 76 | (38.4%)  3 | | 320 | | (48.9%) | | |
|  | | |  |  | |  |  | |  | |  | | |
| **Tumor grade** | | | | | |  | | |  | | | | |
| Missing data | | | 5 |  | | 1 | | | N/A | | |  | |
| Grade 1 | | | 14 | (6.6%) | | 30 | (15.2%) | |  |  |  |  |  |
| Grade 2 | | | 59 | (28.0%) | | 83 | (42.1%) | |  |  |  |  |  |
| Grade 3 | | | 138 | (65.4%) | | 84 | (42.6%) | |  |  |  |  |  |
|  | | |  |  | |  |  | |  | |  | | |
| **Tumor stage** | | | | | |  | | |  | | | | |
| Missing data | | | N/A |  | | N/A |  | | 8 | |  | | |
| Stage I | | |  |  |  |  |  | | 125 | | (19.1%) | | |
| Stage II | | |  |  |  |  |  | | 385 | | (59.0%) | | |
| Stage III | | |  |  |  |  |  | | 143 | | (21.9%) | | |
|  | | |  |  | |  |  | |  | |  | | |
| **Breast cancer histological subtype** | | | | | |  | | |  | | | | |
| Missing data | | | 2 |  | | N/A |  | | 1 | |  | | |
| IDC | | | 37 | (17.3%) | |  |  | | 501 | | (75.9%) | | |
| IDC + DCIS | | | 141 | (65.9%) | |  |  | | N/A | |  | | |
| ILC | | | 4 | (1.9%) | |  |  | | 101 | | (15.3%) | | |
| ILC + LCIS | | | 4 | (1.9%) | |  |  | | N/A | |  | | |
| Others | | | 28 | (13.1%) | |  |  | | 58 | | (8.8%) | | |
|  | | |  |  | |  |  | |  | |  | | |
| **ER status** | | | | | |  | | |  | | | | |
| Missing data | | | 2 |  | | 0 |  | | 42 | |  | | |
| Negative | | | 70 | (32.7%) | | 31 | (15.7%) | | 153 | | (24.7%) | | |
| Positive | | | 144 | (67.3%) | | 167 | (84.3%) | | 466 | | (75.3%) | | |
|  | |  | | | |  | | |  | | | | |
|  | |  | | | |  | | |  | | | | |
|  | |  | | | |  | | |  | | | | |

|  | **TTBD** | | | **IGR-2** | | **TCGA** | | |
| --- | --- | --- | --- | --- | --- | --- | --- | --- |
| **PR status** | | | |  | |  | | |
| Missing data | | 2 |  | 0 |  | 46 |  | |
| Negative | | 117 | (54.7%) | 72 | (36.4%) | 205 | (33.3%) | |
| Positive | | 97 | (45.3%) | 126 | (63.6%) | 410 | (66.7%) | |
|  | |  |  |  |  |  |  | |
| **HER2 status** | | | |  | |  | | |
| Missing data | | 16 |  | 0 |  | 226 |  | |
| Negative | | 127 | (63.5%) | 185 | (93.4%) | 342 | (78.6%) | |
| Positive | | 73 | (36.5%) | 13 | (6.6%) | 93 | (21.4%) | |
|  | |  |  |  |  |  |  | |
| **Breast cancer subtype** | | | |  | |  | | |
| Missing data | | 16 |  |  | | 235 | |  |
| ER+ PR+/- HER2- | | 87 | (43.5%) | N/A |  | 265 | (62.2%) | |
| ER+/- PR+/- HER2+ | | 73 | (36.5%) |  |  | 93 | (21.8%) | |
| ER- PR- HER2- | | 40 | (18.5%) |  |  | 68 | (16.0%) | |
|  | |  |  |  |  |  |  | |
| Missing data | | N/A |  |  |  | N/A |  | |
| HR+ HER2- | |  |  | 159 | (80.3%) |  |  | |
| HER2+ | |  |  | 13 | (6.6%) |  |  | |
| HR- HER2- | |  |  | 26 | (13.1%) |  |  | |
|  | |  |  |  |  |  |  | |
| **Breast cancer molecular subtype** | | | |  | |  | | |
| Missing data | | N/A |  | N/A |  |  |  | |
| Normal-like | |  |  |  |  | 19 |  | |
| Luminal A | |  |  |  |  | 247 | (38.5%) | |
| Luminal B | |  |  |  |  | 200 | (31.2%) | |
| HER2 enriched | |  |  |  |  | 76 | (11.8%) | |
| Basal-like | |  |  |  |  | 119 | (18.5%) | |
|  | |  |  |  |  |  |  | |
| **Chemotherapy** | |  |  |  |  |  |  | |
| Missing data | | N/A |  | 0 |  | N/A |  | |
| No adjuvant | |  |  | 76 | (38.4%) |  |  | |
| Adjuvant | |  |  | 122 | (61.6%) |  |  | |
|  | |  |  |  |  |  |  | |
| **Radiotherapy** | |  |  |  |  |  |  | |
| Missing data | | N/A |  | 0 |  | N/A |  | |
| No adjuvant | |  |  | 38 | (19.2%) |  |  | |
| Adjuvant | |  |  | 160 | (80.8%) |  |  | |
|  | |  |  |  |  |  |  | |
| **Hormonotherapy** | |  |  |  |  |  |  | |
| Missing data | | N/A |  | 1 |  | N/A |  | |
| No adjuvant | |  |  | 29 | (14.7%) |  |  | |
| Adjuvant | |  |  | 168 | (85.3%) |  |  | |

N/A, not applicable, when information was not available; HR, Hormonal Receptors. * Small tumor < 30mm (TTBD) or < 20 mm (TCGA, IGR-2); large tumor ≥ 30 mm (TTBD) or ≥ 20 mm (TCGA, IGR-2)

**Supplementary table 2 –** **Multivariate Cox regression analyses of *FBL* mRNA expression and gold standard prognostic factors with distant disease-free survival in IGR-2 series**

|  | **Distant disease-free survival** | | |
| --- | --- | --- | --- |
| **Variables** | **HR** | **CI 95%** | **P-value** |
| ***FBL*** | | | |
| Intermediate | 1.00 |  |  |
| Low | 3.89 | [1.07-14.11] | **0.0390*** |
| High | 3.92 | [1.11-13.91] | **0.0344*** |
| **Tumor size** | | | |
| [7.0-19.7] | 1.00 |  |  |
| [19.7-25.0] | 3.34 | [0.65-17.20] | 0.1497 |
| [25.0-85.0] | 5.91 | [1.15-30.41] | **0.0340*** |
| **Lymph node invasion status** | | | |
| N = 0 | 1.00 |  |  |
| N ≥ 1 | 2.56 | [0.77-8.59] | 0.1268 |
| **Tumor grade** | | | |
| 1 | 1.00 |  |  |
| 2 | 3.28 | [0.37-29.22] | 0.2880 |
| 3 | 5.11 | [0.56-46.70] | 0.1481 |
| **Breast cancer subtype** | | | |
| HR+ HER2- | 1.00 |  |  |
| HER2+ | 0.89 | [0.17-4.66] | 0.8890 |
| HR- HER2- | 1.65 | [0.31-8.85] | 0.5594 |
| **Age (years)** | | | |
| [22.6-56.5] | 1.00 |  |  |
| [56.5-73.2] | 0.26 | [0.07-1.01] | 0.0524 |
| [73.2-93.4] | 2.29 | [0.87-5.99] | 0.0922 |
| **Chemotherapy** |  |  |  |
| No adjuvant | 1.00 |  |  |
| Adjuvant | 0.80 | [0.28-2.32] | 0.6823 |
| **Radiotherapy** |  |  |  |
| No adjuvant | 1.00 |  |  |
| Adjuvant | 2.23 | [0.43-11.52] | 0.3392 |
| **Hormonotherapy** |  |  |  |
| No adjuvant | 1.00 |  |  |
| Adjuvant | 2.03 | [0.42-9.66] | 0.3755 |

HR, Hazard Ratio; CI 95%, 95% of Confidence Interval

**Supplementary table 3 – Characteristics of patients from the CLB-1 and IGR-1 breast cancer series**

|  | **CLB-1** | | | | **IGR-1** | | | |
| --- | --- | --- | --- | --- | --- | --- | --- | --- |
|  | | | (n = 389) | | (n = 1759) | | | |
| **Age** | | | | |  |  |  | |
| Min | | | 26 |  |  | 27 |  | |
| Max | | | 91 |  |  | 95 |  | |
| Median | | | 58.3 |  |  | 60.0 |  | |
|  | | | | |  |  |  | |
| **Tumor size** | | | | |  |  |  | |
| Missing data | | | 0 |  |  | 0 |  | |
| Small (< 30mm) | | | 275 | (70.7%) |  | 1481 | (84.2%) | |
| Large (≥ 30 mm)  ( | | | 114 | (29.3%) |  | 278 | (15.8%) | |
|  | | | | |  |  |  | |
| **Lymph node invasion status** | | | | |  |  |  | |
| Missing data | | | 1 |  |  | 2 | | |
| N = 0 | | | 165 | (42.5%) |  | 1158 | (65.9%) | |
| N ≥ 1 | | | 223 | (57.5%) |  | 599 | (34.1%) | |
|  | | | | |  |  |  | |
| **Tumor grade** | | | | |  |  |  | |
| Missing data | | | 0 |  |  | 2 | | |
| Grade 1 | | | 65 | (16.7%) |  | 441 | (25.1%) | |
| Grade 2 | | | 189 | (48.6%) |  | 815 | (46.4%) | |
| Grade 3 | | | 135 | (34.7%) |  | 501 | (28.5%) | |
|  | | | | |  |  |  | |
| **ER status** | | | | |  |  |  | |
| Missing data | | | 0 |  |  | 0 |  | |
| Negative | | | 50 | (12.9%) |  | 257 | (14.6%) | |
| Positive | | | 339 | (87.1%) |  | 1502 | (85.4%) | |
|  | | |  |  |  |  |  | |
| **PR status** | | | | |  |  |  | |
| Missing data | | | 0 |  |  | 1 |  | |
| Negative | | | 98 | (25.2%) |  | 554 | (31.5%) | |
| Positive | | | 291 | (74.8%) |  | 1204 | (68.5%) | |
|  | | |  |  |  |  |  | |
| **HER2 status** | | | | |  |  |  | |
| Missing data | | 0 | |  |  | 2 | | |
| Negative | | 358 | | (92.0%) |  | 1575 | (89.6%) | |
| Positive | | 31 | | (8.0%) |  | 182 | (10.4%) | |
|  | | | | |  |  |  | |
| **Breast cancer subtype** | | | | |  |  |  | |
| Missing data | | 0 | |  |  | | |  |
| Luminal A | | 207 | | (53.2%) | N/A | |  | |
| Luminal B | | 132 | | (33.9%) |  |  |  | |
| HER2 enriched | | 12 | | (3.1%) |  |  |  | |
| Triple Negative | | 38 | | (9.8%) |  |  |  | |
|  | |  | |  |  | |  | |
| Missing data | |  | |  | 2 | |  | |
| ER+ HER2- | | N/A | |  | 1390 | | (79.1%) | |
| ER- HER2+ | |  |  |  | 182 | | (10.4%) | |
| ER- HER2- | |  |  |  | 185 | | (10.5%) | |

**Supplementary References**

1. Ciriello G, Gatza ML, Beck AH, Wilkerson MD, Rhie SK, Pastore A, et al. Comprehensive Molecular Portraits of Invasive Lobular Breast Cancer. Cell. 2015;163:506–19.

2. Maire V, Baldeyron C, Richardson M, Tesson B, Vincent-Salomon A, Gravier E, et al. TTK/hMPS1 is an attractive therapeutic target for triple-negative breast cancer. PLoS One. 2013;8:e63712.

3. Bourdon J-C, Khoury MP, Diot A, Baker L, Fernandes K, Aoubala M, et al. p53 mutant breast cancer patients expressing p53$γ$ have as good a prognosis as wild-type p53 breast cancer patients. Breast Cancer Res. 2011;13:R7.

4. Marcel V, Ghayad SE, Belin S, Therizols G, Morel AP, Solano-Gonzàlez E, et al. P53 Acts as a Safeguard of Translational Control by Regulating Fibrillarin and rRNA Methylation in Cancer. Cancer Cell. 2013;24:318–30.

5. Nguyen Van Long F, Lardy-Cleaud A, Bray S, Chabaud S, Dubois T, Diot A, et al. Druggable Nucleolin Identifies Breast Tumours Associated with Poor Prognosis That Exhibit Different Biological Processes. Cancers (Basel) [Internet]. 2018;10:390. Available from: http://www.mdpi.com/2072-6694/10/10/390

6. Livak KJ, Schmittgen TD. Analysis of relative gene expression data using real-time quantitative PCR and the 2-ΔΔCT method. Methods. 2001;25:402–8.

7. Erales J, Marchand V, Panthu B, Gillot S, Belin S, Ghayad SE, et al. Evidence for rRNA 2′-O-methylation plasticity: Control of intrinsic translational capabilities of human ribosomes. Proc Natl Acad Sci [Internet]. 2017;114:12934–9. Available from: http://www.pnas.org/lookup/doi/10.1073/pnas.1707674114

8. Huang DW, Sherman BT, Lempicki RA. Systematic and integrative analysis of large gene lists using DAVID bioinformatics resources. Nat Protoc. 2008;4:44–57.

9. Gourgou-Bourgade S, Cameron D, Poortmans P, Asselain B, Azria D, Cardoso F, et al. Guidelines for time-to-event end point definitions in breast cancer trials: results of the DATECAN initiative (Definition for the Assessment of Time-to-event Endpoints in CANcer trials). Ann Oncol Off J Eur Soc Med Oncol [Internet]. 2015;26:2505–6. Available from: http://www.ncbi.nlm.nih.gov/pubmed/26467471
