# Supplementary figures and images for: Low level of Fibrillarin, a ribosome biogenesis factor, is a new independent marker of poor outcome in breast cancer
Source: BMC Cancer. 2022 May 11;22:526. doi: 10.1186/s12885-022-09552-x (PMC9092774; doi:10.1186/s12885-022-09552-x)

Original data - 1

Uncropped western blot from Supplementary Figure 3B-C

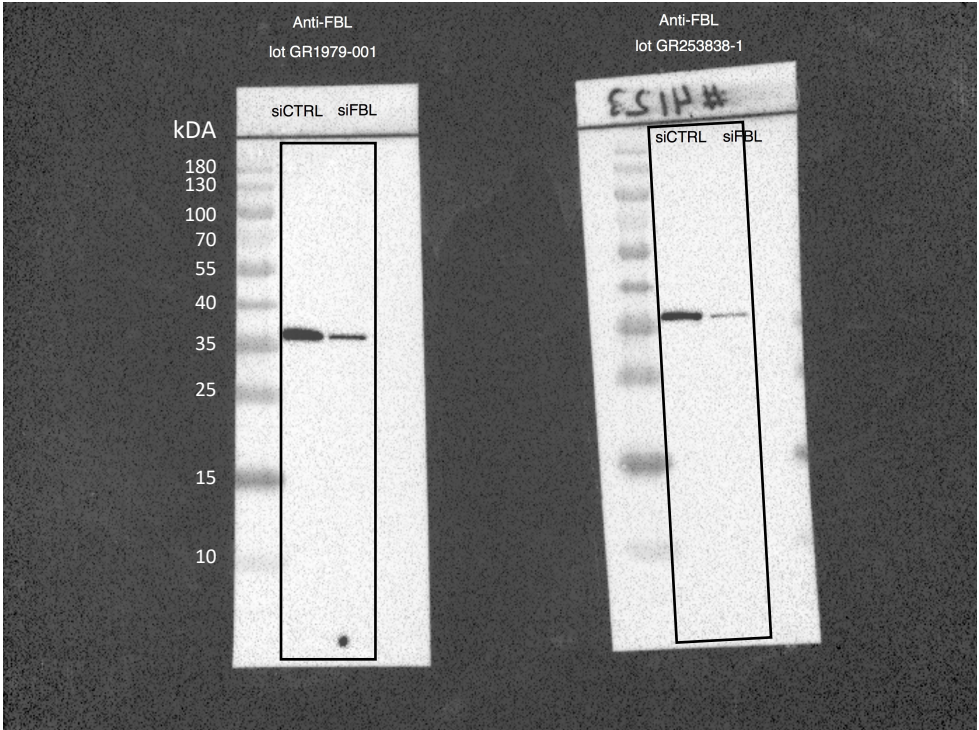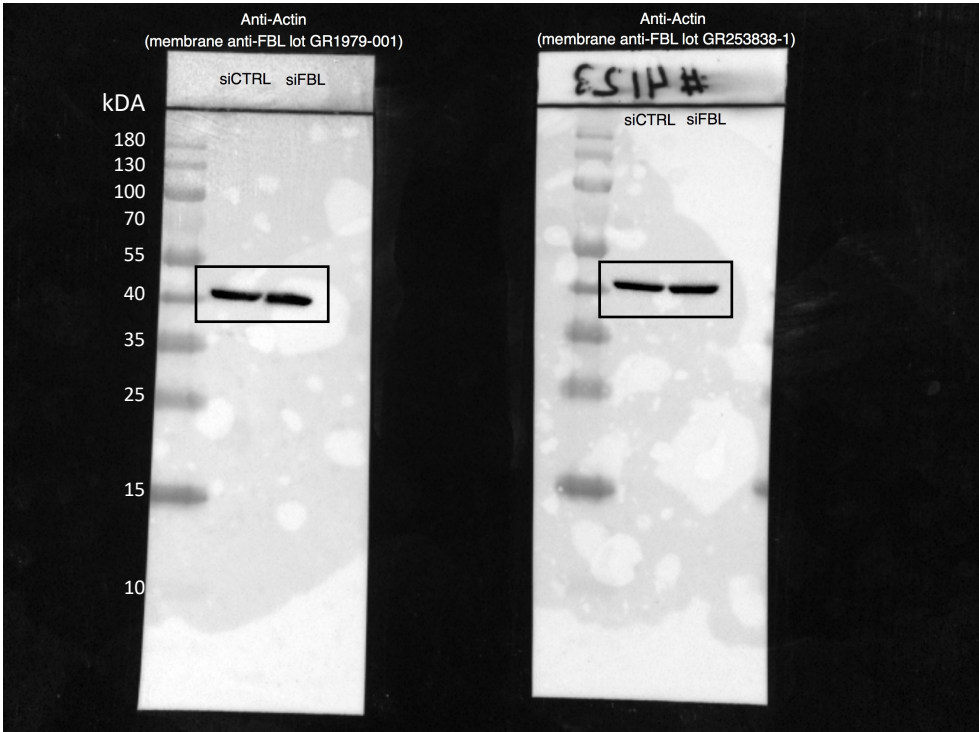

Supplement: Supplementary file 1 — Additional file 1: Supplementary Methods. Supplementary figure 1. Association between FBL mRNA levels and patient survival. Supplementary figure 2. Improvement of breast cancer patient stratification derived from tumor size and lymph node invasion status using FBL mRNA expression. Supplementary figure 3. Validation of FBL antibodies used for FBL immunostaining. Supplementary figure 4. Association between FBL immunostaining and patient survival in CLB-1 and IGR-1 series. Supplementary figure 5. Characterization of the three tumor groups expressing different FBL mRNA levels. Supplementary table 1. Characteristics of patients from TTBD, IGR-2 and TCGA RNA breast cancer series. Supplementary table 2. Multivariate Cox regression analyses of FBL mRNA expression and gold standard prognostic factors with distant disease-free survival in IGR-2 series. Supplementary table 3. Characteristics of patients from the CLB-1 and IGR-1 breast cancer series. [file 12885_2022_9552_MOESM1_ESM.zip › 06-2021-04-23_Full_WB_Cancers_Nguyen.pdf]
